# Advancing workforce development and scientific collaboration: A novel resource for biostatistical education

**DOI:** 10.1017/cts.2024.694

**Published:** 2025-01-02

**Authors:** Emily Slade, Claudine T. Jurkovitz, Shari Messinger, Robert A. Oster, Gina-Maria Pomann, Sandra L. Taylor, Ann M. Brearley

**Affiliations:** 1 Department of Biostatistics, University of Kentucky, Lexington, KY, USA; 2 Institute for Research in Equity and Community Health, ChristianaCare Health Services Inc., Wilmington, DE, USA; 3 Division of Biostatistics, Department of Public Health Sciences, Miller School of Medicine, University of Miami, Coral Gables, FL, USA; 4 Division of General Internal Medicine and Population Science, Department of Medicine, University of Alabama at Birmingham, Birmingham, AL, USA; 5 Department of Biostatistics and Bioinformatics, Duke University, Durham, NC, USA; 6 Department of Public Health Sciences, School of Medicine, University of California, Davis, CA, USA; 7 Division of Biostatistics and Health Data Science, School of Public Health, University of Minnesota, Minneapolis, MN, USA

**Keywords:** Biostatistics, training, education, workforce development, statistical literacy

## Abstract

A clinical and translational scientist (CTS) often seeks to increase their knowledge of statistical topics to effectively conduct biomedical research studies. A common method for obtaining this knowledge is through existing online educational materials that are suggested by a biostatistical collaborator or identified by the CTS. However, the volume of available educational materials on diverse statistical topics makes the task of identifying high-quality educational resources at an appropriate level challenging and time consuming for CTSs and collaborative biostatisticians. In response to these challenges, the Biostats4You website was created, where existing online educational materials for a variety of statistical topics are vetted to identify those most appropriate for CTSs. In this manuscript, we describe the resource review process, provide information about statistical topics and resources currently available, and make recommendations for how CTSs and collaborative biostatisticians can utilize the Biostats4You website to improve training, mentoring, and collaborative research practices.

## Introduction

Statistical knowledge is critical for a clinical and translational scientist (CTS) to appropriately design, conduct, and interpret research, as well as to ensure the rigor of scientific studies. An understanding of statistical methods is essential not only to minimize risks to human subjects but also to ensure that research findings are valid, reliable, and generalizable. Previous work defined 24 statistical competencies that should be included in graduate curricula in health science fields [1–3]. However, gaps in learning have been identified, especially for specialized statistical competencies, suggesting that many CTSs may lack the necessary tools to critically evaluate research findings or apply advanced methods in their own studies [4]. Furthermore, a 2007 survey of medical students, physicians, and clinical teaching faculty found that clinicians across levels of training recognized the importance of understanding statistical concepts but reported low perceived knowledge in these areas [5]. This lack of statistical literacy is common across many medical fields [6–8] and nonmedical fields [9].

Research on the training of CTSs, including both clinicians and non-clinicians, underscores the need for tailored approaches to statistical education. Specifically, Oster et al. contend that statistical coursework for a CTS trainee should be customized to the learner’s background and career goals, which may vary widely between disciplines and specific research contexts [3]. This suggests that a flexible, learner-centered approach to statistical education could help support CTSs in acquiring the appropriate skills for their work. Furthermore, given the increasing complexity of research questions and data sources, there is a need to equip CTSs with tools and resources that not only reinforce foundational knowledge but also help them acquire more advanced statistical competencies.

Collaborative biostatisticians play critical roles on clinical and translational research teams. We refer to a “collaborative biostatistician” as any quantitative expert with a consultative or collaborative role in a clinical or translational research study, ranging from giving brief advice to being integrated as a team scientist throughout all phases of the study. While providing statistical expertise is a primary function, educating CTSs in statistical concepts is an important contribution, ensuring and enhancing research quality. Tobi et al. include “informal teaching” as one of the seven core tasks performed by a statistical consultant, such as providing an “extended explanation of a logistic regression model to a new client” [10]. In 2007, Deutsch et al. studied the activities that took place in 237 statistical consultations across four academic research institutions and found that 78% of the consultations included an educational component [11]. In addition to statistical education occurring in one-on-one consultations, Welty et al. highlight that collaborative biostatisticians play an important role in meeting the statistical educational needs of CTSs through hosting seminars, teaching formal classes, and providing ongoing, informal education in long-term collaborations [12].

CTSs often seek statistical consultation or collaboration for one of two reasons: (1) their limited statistical knowledge or (2) guidance in more advanced statistical techniques beyond their foundational level of knowledge [11]. In the case of (1), it can be time-prohibitive for a collaborative biostatistician to individually educate CTSs on a wide range of foundational statistical topics. In the case of (2), the CTS may be seeking guidance about a specialized statistical topic that is not in the expertise of the collaborative biostatistician. In both cases, collaborative biostatisticians often turn to sharing educational materials with the CTS to review on their own time. There exists a vast range of educational materials on statistical topics available online. The volume of educational materials available on a breadth of statistical topics makes it challenging and time consuming for collaborative biostatisticians and CTSs to identify high-quality educational materials at an appropriate technical level.

To address this challenge, we created the Biostats4You website with the goal of curating a collection of high-quality, freely available educational materials on statistical topics that are tailored to the needs of CTSs. By completing the time-consuming tasks of scouring and reviewing the vast array of educational materials available on the internet and organizing materials in a centralized location, the Biostats4You website facilitates biostatistical education of CTSs and enhances the capacity of collaborative biostatisticians. By improving efficiency in the research process, the use of the Biostats4You website is also valuable to translational science. This manuscript provides an overview of the Biostats4You website (including the format, management, processes, and usage) as well as guidance for collaborative biostatisticians and CTSs in using the Biostats4You website to improve collaborations and research quality.

## The Biostats4You initiative

The Biostats4You website was started by members of the Biostatistics, Epidemiology, and Research Design Special Interest Group (BERD SIG), part of the Association for Clinical and Translational Science (ACTS). Seven collaborative biostatisticians and one CTS from eight different institutions volunteered to form the working group in February 2019. Of the eight institutions represented in the working group, seven were universities with National Institutes of Health (NIH) Clinical and Translational Science Awards Programs, and one was a similar consortium of academic medical centers and universities funded by an NIH Institutional Development Award (IDeA). The initial intent of the working group was to identify strategies to improve efficiency in identifying existing statistical training resources that are of high quality and appropriate for CTSs. From our collective experiences, we reasoned that CTSs would be well-served by a small number of carefully selected and reviewed resources chosen to be most suitable for this audience and of verified accuracy and quality. To address this goal, we created a publicly available website, Biostats4You, which can be found at https://biostats4you.umn.edu [13]. To our knowledge, there are no similar repositories that aim to curate a small number of carefully selected and reviewed statistical resources specifically for CTSs. The original audience for Biostats4You was clinical researchers and professionals who wish to learn more about biostatistics; however, we found that the site is broadly useful for CTSs across biomedical fields.

The structure and initial content of Biostats4You were developed over the next year, and the site was launched on February 1, 2020. The process for selecting content for Biostats4You is described in the next section. The website architecture was developed and is owned and maintained by the University of Minnesota, through its Clinical and Translational Science Institute, as part of its service to the national network of clinical and translational science organizations and to the field of clinical and translational research generally.

The website content is developed, monitored, and updated by the Biostats4You Board of Directors, which meets monthly to set policies and review criteria, choose topics to cover, and oversee the review and approval process for adding new resources and updating topics. The initial Biostats4You workgroup comprised the inaugural board of directors, and board members have been gradually replaced since then. As of 2024, the Board consists of 10 members, including 9 collaborative biostatisticians and 1 CTS. Board members are staff or faculty who are self-nominated or nominated by other BERD SIG members and generally serve two-year terms.

Information about the Biostats4You site has been disseminated through several channels. During monthly virtual BERD SIG meetings, SIG members have been encouraged to utilize Biostats4You and/or share information about it within their institutions. Many BERD units have posted links to the Biostats4You site on their websites. When new topics are added to the site, announcements are sent through the BERD SIG distribution list and posted in relevant community forums such as the American Statistical Association’s Consulting Section and Teaching Statistics in the Health Sciences Section forums. Flyers advertising the resource have also been made available to CTSs at the ACTS’s “Translational Science” conference and at the Joint Statistical Meetings in recent years.

## Biostats4You educational resources

The resources selected for Biostats4You can be in a variety of formats, as long as they are freely available. Selected resources are arranged into topics, such as “Sample Size Calculators” and “Data Collection and Management.” Topics on the Biostats4You site cover various stages of the research lifecycle, with some focused on specific stages (such as study planning, statistical analysis, and interpretation), while others span multiple stages. Within each topic area, we aim to post three to six resources in a variety of lengths, formats, and depth of coverage. Resources in different formats help to meet the needs of different types of learners, and a mix of short and long resources allows users to choose based on how much time they want to spend. Nearly all of the resources on Biostats4You are links to other sites and are not actually stored within Biostats4You.

Topic areas can be suggested to the Board by anyone, directly or via the website. Board members collectively decide which topics should be added to the Biostats4You site. Once a topic is chosen, a board member is assigned to manage its development as a “topic manager.” The topic manager sources potentially appropriate resources from colleagues, friends, internet searches, library searches, etc. Each candidate resource is then reviewed by at least two reviewers, ideally one collaborative biostatistician and one CTS, in addition to the topic manager (board member). Reviewers are selected from a pool of volunteers; the only criterion for being part of the Biostats4You reviewer pool is that one must be actively working as a collaborative biostatistician or a CTS. The process of collecting and reviewing resources is managed using a REDCap database located at the University of Minnesota [14].

The review criteria for resources are shown in Table 1. Each reviewer is asked via a REDCap survey to rate the candidate resource on each criterion and provide a few sentences giving their overall impression and recommendation. The topic manager considers the reviews, as well as their own assessment, in recommending which resources to post for that topic. The Biostats4You site is checked for broken links multiple times per year, and the Board plans to review existing topics and resources every five years after the initial posting.

**Table 1.** Review criteria for resources to be included on the Biostats4You website

Criteria	For all resources	For online calculators
Accessibility	“In what way is the resource accessible?,” with choices of “Freely available with no login required,” “Freely available with site registration required,” and “Behind a paywall.” The resource must be freely available to anyone, and we prefer that site registration (even when free) is not required, since it adds an additional barrier to a potential user. We do not post resources that are behind a paywall.	In addition to the points for all resources, the calculator should run directly from the web browser and not require downloading anything (e.g., applets, software packages). We viewed the need to download something as a barrier to ease of use. We also downrated sites that contained advertisements, particularly pop-up ads, as those would be expected to be annoying to users.
Audience	“To what degree is the technical detail appropriate for a clinical or translational scientist?,” rated on a five-point scale from “Too basic” to “Too technical.” The resource must be written at a technical level that is appropriate for clinical and translational scientists.	No additional criteria.
Clarity	“How clearly does the resource present the material?,” rated on a five-point scale from “Not clear” to “Exceptionally clear.”	In addition to the points for all resources, reviewers checked that the calculator clearly distinguished what the user needs to enter as inputs from what the program is calculating as output and clearly described the output. For example, for sample size calculators, we downrated sites that requested the user to input beta instead of power or that allowed nonsensical inputs such as power values outside of the range of 0–1. Overall, we look for calculators that are easy to use and difficult to misuse.
Conciseness	“How efficiently is the material presented?,” rated on a five-point scale from “Skips important details” to “Includes irrelevant or spurious information.” The resource must explain the topic concisely without leaving out important details. Clinical/translational scientists don't have time to wade through a lot of irrelevant or spurious information.	Not applicable.
Independence	“To what degree does the resource stand on its own?,” rated on a five-point scale from “Resource requires readers to be familiar with material outside the resource” to “Resource is self-contained; no outside information is needed.”	No additional criteria.
Accuracy	“To what degree is the material accurate?,” rated on a five-point scale from “There are disqualifying errors in the resource” through “There are errors/oversimplifications but they are not disqualifying” to “Resource is accurate.” The resource should be accurate and correct. It may have a few oversimplifications, but it should not have any gross disqualifying errors.	In addition to the points for all resources, reviewers checked the accuracy of calculators with a few key test cases.
Transparency	Not applicable – included only for review of online calculators.	We uprated sites which stated the assumptions and methods used in their calculations, ideally somewhere unobtrusive such as a footnote or a link to another page.
Reputation	“What is your opinion of the source?,” rated on a five-point scale from “Unknown reputation” to “Highly reputable.” The person, people, or organization responsible for creating the resource must be reputable and trustworthy.	No additional criteria.
Overall Assessment	Rated on a five-point scale from “Do not recommend” through “Neutral” to “Highly recommend.”	No additional criteria.

As of 2024, Biostats4You contains 45 resources covering twelve topic areas. The 12 topics on Biostats4You are described briefly in Table 2. Table 3 shows the number of unique pageviews during the past year, as obtained from Google Analytics. Sample size calculators have consistently been the most popular topic since the site was launched. However, it should be noted that some topics and resources have been on the site longer than others (see Table 3 for duration). The top 15 most-viewed individual resources over the last year from June 1, 2023, to June 1, 2024, were obtained from Google Analytics and are shown in Table 4.

**Table 2.** Description of Biostats4You topics, their purpose, and the range of resources within each

Topic	Description of resources
Finding and Working with a Biostatistician	These six resources provide guidance to clinical and translational scientists (CTSs) on how to find and work with a biostatistician or other quantitative expert. 1) A webpage on what to expect in consulting or collaboration 2, 3) A blog post and a 10-minute video on keys to successful collaboration 4) A peer-reviewed article describing how a collaboration proceeds through different phases of a study 5) A slide deck from a lecture on how clinicians and biostatisticians work together on a study from the planning stage through data collection to publication, illustrated with real-world examples (and cartoons!) 6) A webpage written by the Biostats4You Board describing some of the places a clinical/translational scientist might find quantitative experts at their own institution
Sample Size Calculators	These three resources help CTSs carry out basic sample size calculations fairly quickly and easily. The three sites collectively cover a range of common situations, including binary, continuous, and time-to-event outcomes, and superiority, equivalence, and noninferiority study designs. 1) stattools.crab.org 2) sample-size.net 3) sealedenvelope.com
Power and Sample Size Concepts	These six resources explain the fundamental concepts of power and sample size, what they depend on, and why they matter. 1) A webpage covering the fundamentals of power and sample size 2) A five-minute YouTube video describing power, type 2 error, and sample size 3) A 35-minute webinar on sample size calculations for grant applications 4–6) Three 1–2-hour-long video lectures, one of which focuses on cluster designs
Study Designs	These three resources provide an overview of the main observational and experimental study designs and the advantages and limitations of each. 1) A webpage describing various type of study designs and how they rank in terms of evidential strength 2) A peer-reviewed article providing an introductory overview of study designs 3) A 45-minute video lecture covering observational and experimental designs
Data Collection and Management	These four resources provide guidance on collecting and managing study data. 1) A peer-reviewed article on the use of spreadsheets for data collection 2) A Data Tips spreadsheet with 25 pages, each demonstrating specific “bad” and “good” ways to enter data 3) The Project REDCap Consortium’s training videos 4) The University of California’s DMPTool, a free online tool for creating a data management plan for grant applications.
Collecting Race and Ethnicity Data	These two resources describe considerations and best practices for collecting and reporting data on race or ethnicity in a study. 1) An editorial published in the Journal of the American Medical Association providing guidance on reporting 2) A 50-minute video lecture on collecting race and ethnicity data
Nonparametric Methods	These four resources describe commonly used nonparametric methods and their advantages and disadvantages. 1, 2) Two peer-reviewed articles published in clinical journals 3) A short reference document from Mayo Clinic’s consulting center 4) A 1-hour recorded lecture describing how to use basic nonparametric statistical methods
Multiple Testing	These three resources explain what multiple testing is, why it is problematic, and how to recognize and control for it. 1) A short six-page peer-reviewed article 2) An 11-minute YouTube video describing how to recognize multiple testing problems in medical research 3) A 14-page educational document from Duke University’s Global Health Institute
Basic Statistical Tests	These two resources describe what the tests are for, when to use them, and how to interpret the results. 1) A 20-minute YouTube video on chi-squared tests 2) A set of webpages on *t*-tests
Hypothesis Tests and p-Values	These five resources explain the concepts involved in carrying out hypothesis tests and correctly interpreting the resulting *p*-values. 1, 2) Two excellent short (7–8 minutes) videos on foundational concepts of hypothesis testing 3) A one-hour recorded lecture covering the use and interpretation of *p*-values 4) A blog post describing how to interpret a “non-significant” *p*-value 5) A three-minute YouTube video on “Why *p*-Values are like Puppies”
Screening and Diagnostic Tests	These five resources explain the key concepts involved in designing, evaluating, and interpreting the results of diagnostic tests. 1, 2) Two peer-reviewed one-page articles by Altman and Bland, one on sensitivity and specificity and the other on positive and negative predictive values 3, 4) Two 12-minute YouTube videos, one on sensitivity and specificity and the other on ROC curves 5) A one-hour recorded lecture that clearly explains all of these concepts as well as AUC
Post-hoc Power Analyses	These two resources explain what post hoc power analyses are and why they are not useful in most cases. 1) A blog post that clearly explains what post hoc power is and why it is (in their words) a “useless statistical concept” 2) A peer-reviewed 2018 article explaining why post hoc power analyses are nonsensical

**Table 3.** The 12 topics currently on the Biostats4You site, in order of unique pageviews during the past year, June 1, 2023–June 1, 2024

Topic	Number of resources	Date posted	Unique pageviews
Sample Size Calculators	3	Feb 2020	1165
Hypothesis Tests and *p*-Values	5	June 2020	432
Power and Sample Size Concepts	6	Feb 2020	347
Nonparametric Methods	4	July 2022	310
Finding and Working with a Biostatistician	6	Feb 2020	262
Screening and Diagnostic Tests	5	Oct 2020	240
Post-hoc Power Analyses	2	Apr 2023	185
Study Designs	3	Dec 2023	179
Multiple Testing	3	Apr 2022	164
Data Collection and Management	4	Feb 2020	155
Basic Statistical Tests	2	Feb 2024	98
Collection of Race and Ethnicity Data	2	Feb 2024	69

**Table 4.** Most-viewed individual resources on the Biostats4You site during the past year, June 1, 2023–June 1, 2024

Resource	Source	Topic	Unique pageviews
Sample size calculators from sealedenvelope.com	Sealed Envelope Ltd (London, UK)	Sample Size Calculators	582
Sample size calculators from sample-size.net	University of California-San Francisco Clinical and Translational Science Institute	Sample Size Calculators	430
“What to Say (and Not Say) when *p* > 0.05?,” blog post	Thomas G. Stewart, PhD, Vanderbilt University Medical Center (Nashville, TN)	Hypothesis Tests and p-Values	282
“Introduction to Power Analysis,” webpage	University of California-Los Angeles Statistical Consulting Group	Power and Sample Size Concepts	189
Sample size calculators from crab.org	Cancer Research and Biostatistics (Seattle, WA)	Sample Size Calculators	153
“Parametric and Nonparametric: Demystifying the Terms,” document	Tanya Hoskin, MS, Mayo Clinic Department of Health Sciences Research (Rochester, MN)	Nonparametric Methods	147
“The Trade-off between Sensitivity and Specificity,” 12-minute YouTube video	Rahul Patwari, MD, Rush Medical College (Chicago, IL)	Screening and Diagnostic Tests	116
“What is Post-hoc Power Anyway?,” blog post	Daniel Lakens, PhD, Eindhoven University of Technology (Netherlands)	Post-hoc Power Analyses	94
“Why Post-hoc Power Analyses are Nonsensical,” published article (2018)	Michael R. Jiroutek, DrPH, Campbell University, and J. Rick Turner, PhD, Statistical Consultant (North Carolina)	Post-hoc Power Analyses	91
“Statistics Review 6: Nonparametric Methods,” published article (2002)	Elise Whitley, University of Bristol and Jonathan Ball, St George’s Hospital Medical School (UK)	Nonparametric Methods	74
“Multiple Comparisons,” 11-minute YouTube video	Steven Grambow, PhD, Duke University School of Medicine (Durham, NC)	Multiple Testing	73
“Evidence-Based Practice: Levels of Evidence and Study Designs,” webpage	Bev Sedlacek, Library Director at Nebraska Methodist College and Nebraska Methodist Health System and Ascension Wisconsin Library Services	Study Designs	63
“An Overview of Clinical Research: The Lay of the Land,” published article (2002)	David A. Grimes, MD, and Kenneth F. Schulz, PhD, Family Health International and University of North Carolina School of Medicine	Study Designs	60
“Nonparametric Statistical Tests for the Continuous Data: the basic concept and the practical use,” published article (2016)	Francis Sahngun Nahm, MD, PhD, Seoul National University’s Bundang Hospital	Nonparametric Methods	58
“Data Collection in Spreadsheets,” published article (2018)	Karl W. Broman, PhD, University of Wisconsin-Madison and Kara H. Woo, MLIS, University of Washington-Seattle	Data Collection and Management	56

## User experience

Biostats4You was originally intended for CTSs who are not statisticians. In the four years since launching the site, however, it has become apparent that it is also a valuable resource for collaborative biostatisticians themselves. The ways in which the site can be useful to both CTSs and collaborative biostatisticians are described below, including examples of use cases for several resources. These use cases were developed from the authors’ own experiences, from experiences shared with them by colleagues, and through additional ideas produced by using generative artificial intelligence.

### For clinical and translational scientists (CTSs)

Consistent with its founding purpose, CTSs can use Biostats4You to improve their understanding of study design and biostatistics. This increased understanding is beneficial to CTSs for conducting high-quality research, critically reviewing manuscripts, and responding to reviewer comments.

#### Training programs

In the context of a training program for CTSs, Biostats4You can be an invaluable resource. For instance, a key component of the training program could be “understanding study design.” CTS trainees could be presented with a research question and then directed to the resources on Biostats4You to identify the most appropriate study design. They could then present their chosen design and justify their choice in a group discussion, fostering a deeper understanding of the various study designs and their applicability.

Another component of the training program could be “critical appraisal of research.” Biostats4You provides resources on evidence-based practice, which could be used to enhance the trainees’ skills in this area. CTS trainees could be given a published research paper and asked to critically appraise it using these resources. This exercise would not only help trainees understand the principles of evidence-based practice but also enable them to critically evaluate the quality and relevance of published research. Through such practical exercises, Biostats4You can be effectively used to train CTSs, helping to equip them with the necessary skills to conduct and evaluate research in their respective fields.

#### Self-teaching (journal club)

In a journal club setting, CTSs could utilize Biostats4You resources to enhance their understanding of biostatistics and its application in medical research. For instance, journal club leaders could select a specific topic or tool from the website, such as sample size calculations, and discuss practical applications of the resources in medical research. The discussion could include a review of the underlying statistical concepts, followed by a demonstration of how to use the tool using real-world data. The group could then discuss the results, their implications, and any potential limitations. The discussion could also link the resources on Biostats4You with current articles that utilize specific methodology. This would help the participants understand the statistical tool better and enable them to critically evaluate its use in published research.

#### Preparation for collaborating with a biostatistician

Before engaging in a collaboration with a biostatistician, a CTS could utilize the resources on Biostats4You to prepare for this collaboration. Specifically, a CTS could review resources in the *Finding and Working with a Biostatistician* topic to learn how to find a biostatistician at their institution, how to build a good working relationship, and what to expect when consulting or collaborating with a biostatistician. The resources in this topic provide further insights into how CTSs and biostatisticians can work together effectively at each phase of a research study, real-world examples of such collaborations, and tips for building a successful long-term collaboration with a biostatistician.

#### Mentoring

Both collaborative biostatisticians and CTSs may recommend Biostats4You to their mentees as a resource to address specific areas for statistical training. Sharing such resources has tremendous value in mentoring of trainees (graduate students, postdocs, K-awardees, and others) and junior faculty in medicine, public health, and other areas of biomedical research. The collaborative biostatistician or CTS might recommend the site overall or specific topics on the site, depending on the needs of their mentee. However, the use of the site should be complemented by follow-up discussions with the mentor to ensure that the mentee fully understands the material and to address any questions or challenges they may have about applying the concept in their specific research context.

### For collaborative biostatisticians

#### Collaborating

Collaborative biostatisticians can use Biostats4You to quickly find resources helpful to non-statistical collaborators. For example, resources in the *Power and Sample Size Concepts* topic can be used to help CTSs understand the sample size recommendations the collaborative biostatistician is making. Resources in the *Data Collection and Management* topic can be shared with CTSs at the beginning of a collaboration, to minimize data cleaning effort. Resources in the *Post-hoc Power Analyses* topic can help collaborators respond more effectively to reviewer requests for post hoc power calculations. Many statistical consulting groups have posted a link to Biostats4You on their own website, to direct current and potential collaborators to appropriate resources.

#### Practice

Collaborative biostatisticians can use Biostats4You resources in their own practice of statistics. For example, they might use the online sample size calculators to double-check their own sample size calculations or to quickly check other people’s sample size calculations while reviewing an article or a staff member or student’s work.

#### Formal teaching

Collaborative biostatisticians can use resources on Biostats4You as supplemental materials in their formal undergraduate- or graduate-level introductory statistics courses. One instructor regularly assigns the “What to Say (and Not Say) when *p* > 0.05?” blog post to her students and has anecdotally observed that her students seem less likely to use incorrect terminology after reading it. Another instructor assigned resources in the *Data Collection and Management* topic as homework for medical students before a lecture on best practices for data collection. Biostats4You has also been used more broadly by course instructors; for example, an instructor of a second-semester statistics course includes Biostats4You in the syllabus as a resource for reviewing introductory topics; her students report that it was very useful.

#### Informal teaching

Collaborative biostatisticians can use resources on Biostats4You for informal teaching such as one-time “introduction to statistics” seminars or medical resident journal clubs. For example, resources from the *Hypothesis Tests and p-Values* topic could be used as training materials for one-hour seminars focused on what hypothesis tests are used for and how to interpret the results. The resources could be shared with attendees in advance, used as the basis of in-person discussion, or shared with attendees as supplemental materials following the seminar. The collaborative biostatistician can have confidence that the resources are trustworthy and are accessible to CTSs. An advantage of sending attendees to Biostats4You instead of to the original source for a given resource is that it enables attendees to look around at other resources on that topic or other topics to further their statistical knowledge.

## Discussion

Biostats4You was created to address the challenge of finding high-quality, accessible statistical education materials for CTSs. The website serves as a centralized resource, providing CTSs with a curated set of materials to enhance their statistical literacy. For collaborative biostatisticians, Biostats4You acts as a tool to expand their capacity to educate CTSs on statistical concepts. By organizing existing resources into one site, Biostats4You saves both CTSs and collaborative biostatisticians time and effort, ensuring that materials are clear, accurate, and sourced from reputable organizations. The use cases presented in this paper further illustrate how both CTSs and collaborative biostatisticians can utilize the site for education and research purposes.

Importantly, Biostats4You should not be used as a substitute for input from a collaborative biostatistician when planning or analyzing a study. The resources on the site are intended to provide foundational statistical knowledge for a CTS that complements tailored input that a collaborative biostatistician provides for a specific research project. It is critical for CTSs to recognize the importance of working with a biostatistician on research studies and that materials on Biostats4You complement this collaboration. Resources provided in the Biostats4You topic on *Finding and Working with a Biostatistician* are helpful for appreciating the value of the collaborative relationship.

While Biostats4You has grown steadily since its launch in 2020, adding two to three new topics per year, there are some ongoing challenges with maintaining and growing the Biostats4You site. It can be challenging to find reviewers for the proposed resources, especially clinical reviewers who are able to provide the perspective of whether the proposed resource would be suitable for a non-statistical audience. The Biostats4You Board has also found it challenging to effectively divide some statistical concepts into reasonably sized topics for organization on the site and to identify resources with the appropriate level of depth. For example, a topic such as “Study Design” could include resources that provide a high-level overview of the different types of study designs. Alternatively, this topic could be divided into several sub-topics such as “Cohort Studies,” “Case Control Studies,” and “Randomized Controlled Trials,” each of which may contain resources that provide more depth into the specific issues related to that type of study. The Biostats4You Board aims to identify a small number (3–6) of resources for each topic in order to provide some variety in the length and modality of resources but prevent substantial duplication of content. However, this structure may need to be refined to accommodate more complex topics in the future.

There are several future directions planned for Biostats4You. First, the Biostats4You Board intends to continue adding new topics on an ongoing basis. Anyone can suggest new topics or provide any other suggestions to the Biostats4You team by emailing bdac@umn.edu. The Board also plans to explore opportunities for soliciting feedback on the site from CTSs, including assessing whether the resources have been helpful in improving their statistical knowledge. Additionally, the Biostats4You Board has considered organizing the existing topics and resources into self-paced “courses” that would be suitable for a trainee, such as a medical resident, to work through to develop a basic foundation of statistical knowledge.

In addition to using Biostats4You, CTSs and collaborative biostatisticians have several avenues for furthering their involvement in the initiative. The Biostats4You Board relies on a network of reviewers to help assess the value of each proposed resource. Both CTSs and collaborative biostatisticians can volunteer to review resources by clicking the “Be a Site Reviewer” link on the Biostats4You homepage. Each resource typically takes less than an hour to review, and reviewers typically receive no more than three review requests per year. Reviewers are recognized annually on the Biostats4You site for their service to the CTS community. Both CTSs and collaborative biostatisticians are welcome to serve on the Biostats4You Board and can reach out to bdac@umn.edu to receive more information about the process for joining the Board for a two-year term. Finally, anyone with access to a statistical consulting/collaboration unit’s or clinical department’s webpage is encouraged to include a link to Biostats4You (https://biostats4you.umn.edu) to help ensure that clinical/translational scientists are aware of the site as an educational resource on statistical topics. With support and input from the CTS research community, Biostats4You can continue to grow as a valuable resource.
